# Quantitative Trait Locus Mapping of Clubroot Resistance and *Plasmodiophora brassicae* Pathotype Banglim-Specific Marker Development in *Brassica rapa*

**DOI:** 10.3390/ijms21114157

**Published:** 2020-06-10

**Authors:** Su Ryun Choi, Sang Heon Oh, Sushil Satish Chhapekar, Vignesh Dhandapani, Chang Yeol Lee, Jana Jeevan Rameneni, Yinbo Ma, Gyung Ja Choi, Soo-Seong Lee, Yong Pyo Lim

**Affiliations:** 1 Molecular Genetics and Genomics Laboratory, Department of Horticulture, College of Agriculture and Life Science, Chungnam National University, Daejeon 34134, Korea; srchoi@cnu.ac.kr (S.R.C.); rederaser64@gmail.com (S.H.O.); sushilchhapekar@gmail.com (S.S.C.); tjwlzkf@naver.com (C.Y.L.); saijeevan7@gmail.com (J.J.R.); mayinbo@126.com (Y.M.); 2 School of Biosciences, University of Birmingham, Birmingham B15 2TT, UK; v.dhandapani@bham.ac.uk; 3 Center for Eco-friendly New Materials, Korea Research Institute of Chemical Technology, Daejeon 34114, Korea; kjchoi@krict.re.kr; 4 Bio-Breeding Institute, 153-60 Sinryeong-ro, Anseong 17544, Korea; sslee0872@hotmail.com

**Keywords:** clubroot disease, resistance mapping, quantitative trait loci (QTL), marker, *Brassica rapa*

## Abstract

Clubroot resistance is an economically important trait in Brassicaceae crops. Although many quantitative trait loci (QTLs) for clubroot resistance have been identified in *Brassica*, disease-related damage continues to occur owing to differences in host variety and constant pathogen variation. Here, we investigated the inheritance of clubroot resistance in a double haploid population developed by crossing clubroot resistant and susceptible lines “09CR500” and “09CR501”, respectively. The resistance of “09CR500” to *Plasmodiophora brassicae* pathotype “Banglim” was controlled as a single dominant gene, with the segregation of resistance and susceptibility being nearly 1:1. *PbBrA08^Banglim^* was identified as having a logarithm of odds value of 7.9–74.8, and a phenotypic variance of 26.0–97.1% with flanking marker “09CR.11390652” in A08. After aligning QTL regions to the *B. rapa* reference genome, 11 genes were selected as candidates. *PbBrA08^Banglim^* was located near *Crr1*, *CRs*, and *Rcr9* loci, but differences were validated by marker analysis, gene structural variations, and gene expression levels, as well as phenotypic responses to the pathotype. Genotyping using the “09CR.11390652” marker accurately distinguished the Banglim-resistance phenotypes in the double haploid population. Thus, the developed marker will be useful in *Brassica* breeding programs, marker-assisted selection, and gene pyramiding to identify and develop resistant cultivars.

## 1. Introduction

The Cruciferae family includes various crops that provide sources of daily vegetables and oils for humans and forage crops for animals worldwide. Clubroot, caused by *Plasmodiophora brassicae*, is a soil-borne contagious disease that leads to decreases in crop productivity, including that of Chinese cabbage and other relatives in Cruciferae.

This biotrophic pathogen infects plants through the hairy roots. Consequently, it is difficult to detect because it occurs underground. In the early stages of infection, the plant’s nutrients are taken away from regulating host cell proliferation, as well as vasculature and phloem differentiation, resulting in delayed growth and withering symptoms [1,2]. However, in the later stages of infection, growth halts after the roots are transformed into thick galls (anatomical changes). These modified roots cannot properly transfer moisture and nutrients, which are inherent to the roots. To overcome *P. brassicae* infections, various crop cultivation methods have been introduced, such as management of soil pH and soil humidity; treatments with trace elements such as a calcium, antagonists, and fungicides; and crop rotations with nonhost crops [3,4,5]. However, it is difficult to carry out the crop cultivation control method every year. Additionally, once fields are infected, they are constantly at risk owing to the longevity of the resting spores [6]. Therefore, the development of resistant cultivars is an appropriate countermeasure to maintain sustainable agriculture and productivity [7,8].

Resistance traits have been introgressed successfully through interspecific hybridization with plants having resistance through conventional breeding processes [9,10,11,12,13,14,15]. At present, many studies have reported various genetic factors associated with resistance in diverse crops species (*Brassica rapa*, *Brassica oleracea*, *Brassica juncea*, *Brassica napus*, *Brassica carinata*, and *Raphanus satives*). In *B. rapa* (A genome), 17 quantitative trait loci (QTLs), including two candidate genes, were reported over the last two decades, and these are located in six chromosomes (A01, A02, A03, A05, A06, and A08) in the *B. rapa* genome: *CRa* [16], *CRb* [13,17], *CRaki* [18], *CRc*, *CRk* [19], *CRd* [20], *Crr1a*, *Crr1b*, *Crr2*, *Crr4* [21,22,23], *Crr3* [24], *PbBa3.1*, *PbBa3.3* [13], *CrrA05* [25], *Rcr1* [26], *Rcr9* [27], and *CRs* [28]. In *B. oleracea* (C genome), nine QTLs were detected in four chromosomes (C2, C3, C5, and C9): *Pb-Bo (Anju)1*–*4* [29]*, CRQTL-YC*, *CRQTL-GN_1*, *CRQTL-GN_2* [30], *Rcr7* [31], *QTL-LG9* [32], and 23 QTLs [33]. In *Brassica nigra* (B genome), *Rcr6* was recently identified in B3 chromosome as a single dominant [34]. 

For the amphidiploids, *B. napus* (AC), several results were reported [35,36,37,38]. Additionally, QTLs have been reported in *R. sativus* [39,40]. This information has been useful for the development of markers for marker-assisted selection [41]. Nevertheless, owing to the rapid adaptation (variation) of pathogens to available cultivars, the continuous breeding of new resistant cultivars is required. 

Next-generation sequencing technologies have been applied to crop genetics and breeding programs as powerful tools that accelerate the pace of marker development and the mapping of associated traits by providing massive quantities of sequence variation data (markers) within a short time [42,43,44,45]. Genomic information enables the identification of sequence variations, which facilitates the development of large numbers of markers. Here, we performed genome sequencing of the resistant inbred *B. rapa* line “09CR500” to develop phenotype-associated markers. 

The objectives of this study were i) the identification of genomic regions harboring *P. brassicae* resistance in the inbred line “09CR500”; and ii) the development of the markers tightly associated with *qPbBrA08^Banglim^* for further use in breeding programs to increase clubroot resistance and for marker-assisted selection.

## 2. Results

### 2.1. Inheritance of Clubroot Resistance in Line 09CR500

**Figure 1:**
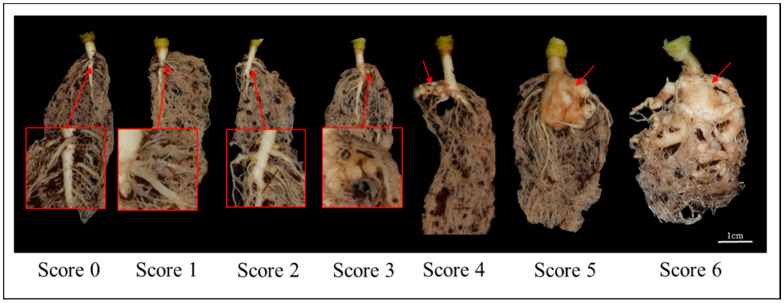
The clubroot disease symptom of roots infected by *Plasmodiophora brassicae*, and the standard of evaluation depending on the severity of root morphology modification. The disease symptom of roots infected and standard of evaluation depending on the severity having lump formation named as club. Symptom was classified on 7 scales from 0 to 6, where 0 = no symptoms; 1 = very slight swelling, usually confined to lateral root; 2 = a few tiny globular nodules on the lateral roots; 3 = several small-sized nodules on lateral roots; 4 = large clubs on lateral roots joining together to form a lump and slight swelling of main roots; 5 = large clubs on main roots; 6 = severe clubs (lumps) formation all over roots and complete change of root morphology.

**Figure 2:**
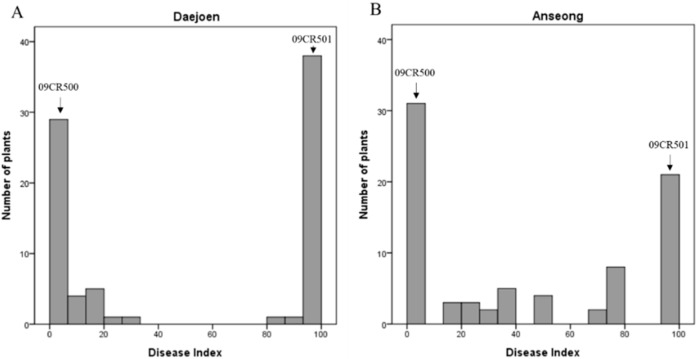
Distribution of mean disease index (DI) investigated over 2 years for clubroot disease in ‘09CR-DH’ population in (**A**) Daejeon and (**B**) Anseong.

Clubroot resistance was evaluated in a doubled haploid (DH) population—including parental lines—over two years. The resistant line “09CR500” represents strong resistance, producing no symptoms in response to the Banglim pathotype, while “09CR501” shows severe symptoms, such as a high level of gall formation (Figure 1, Table S1). The F_1_ also presented the resistant phenotype. In the DH population, phenotypes ranged from “no symptoms” to “severe clubbing over all roots”. The overall phenotypic variation in the DH population exhibited a graph somewhat biased to the both ends (Figure 2). The segregation of resistance to susceptibility was close to a 1:1 ratio. This suggested that the resistance of ‘09CR500” was controlled by a single dominant gene (Table 1).

### 2.2. Whole-Genome Sequencing Analysis and Genetic Map Construction

We used previously reported markers in the construction of a genetic map with the ‘09CR.DH’ population (Table S2). To saturate the map, the resistant “09CR500” line’s genome was sequenced using Illumina HiSeq2500 (BGI Co. Ltd., China) with paired-ends and having an average insert size of ~500 bp. A total of 25.32 Gb of sequence (202.58 M reads) data was produced. After removing adapter reads and low-quality reads (unknown bases, N>5%), 23.18 Gb (202.11 M reads) remained, of which 92.19% were qualified as clean reads on the basis of Q30 (Table S3). The genome coverage was 43.81-fold, and ~94.10% of sequences were mapped (aligned) to the reference genome (v1.2), with an average depth of 87.06×. We predicted sequence variants in two genomes. Among the 2,401,195 single nucleotide polymorphisms (SNPs), only 77.8% was homozygous, even though ‘09CR500” is a DH line in which, theoretically, all the SNPs should be homozygous. There were 28,200 nonsynonymous SNPs in the coding region. In total, 595,678 insertions and deletions (InDel) were identified, with 44,291 being located in the coding region (Table S4).

A total of 808 primers were synthesized and used in screening for polymorphisms between parental lines, and 33.8% of the markers represented polymorphisms. Among diverse marker types, the primers designed with InDel variations as targets identified 68.75% of the polymorphisms (Table S2). All the primer and plant resource information is available in Table S2. Linkage map construction was performed using JoinMap v.4.1. Finally, 222 markers were assigned to 10 linkage groups (LGs), designated as A01–10. The total length of the genetic map was 731.7 cM, with an average distance of 4.4 cM between adjacent markers. The lengths of the LGs ranged from 29.3 for A01 to 131.2 cM for A09. The variation in the number of markers per chromosome ranged from 6 (A01) to 84 (A08) (Table S5). In the genetic map, most of the LGs had marker orders that were collinear to the physical map, with the exceptions of A07, A08, and A10.

### 2.3. QTL Identification and Marker Development

**Figure 3:**
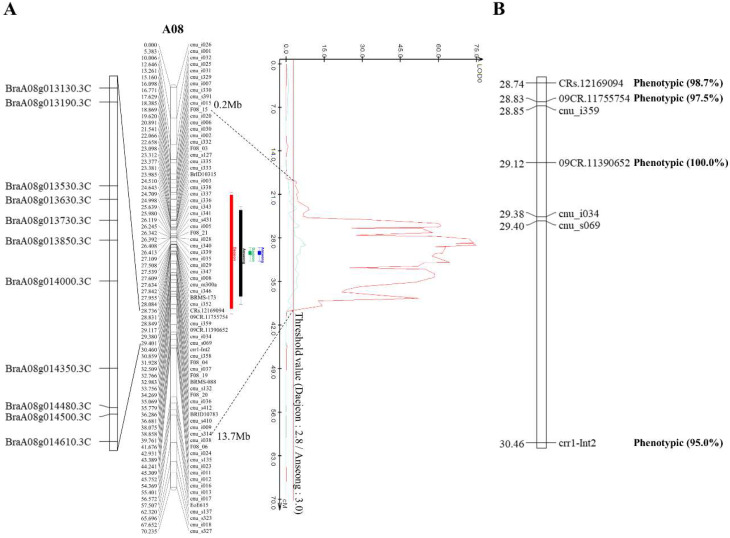
The linkage map of chromosome A08 and QTL, *PbBrA08^Banglim^*, for clubroot resistance traits to the *Plasmodiophora brassicae* Banglim pathotype in doubled haploid population of *B. rapa*. (**A**) The *PbBrA08^Banglim^* in the genetic map and candidate genes. In genetic map, genetic distances are shown on the left side of linkage group as centi-morgans (cM), and the markers are located on the right side of linkage group. On the right side of genetic map is QTL result. On the left side of map represented the candidate genes are represented, located in the overlapping detected QTL region analyzing WinQTL cartographer 2.5 and IciMapping 4.1. The rectangular bars of red and black color represented the QTL region, analyzed by WinQTL cartographer 2.5 and discovered in year replicates, respectively. The green and blue color bar show QTL detected by IciMapping 4.1. (**B**) shows closely linked markers to the resistance loci to *Plasmodiophora brassicae* Banglim pathotype. The value inside of parenthesis represented the identification ratio of phenotype by markers, respectively.

To identify the genomic regions involved in the clubroot resistant phenotype, we analyzed the QTLs using WinQTL cartographer 2.5 and IciMapping 4.1 software. The WinQTL cartographer indicated two significant QTLs, named “*PbBrA08^Banglim^*-1” and “*PbBrA08^Banglim^*-2”, on the A08 chromosome in annual replications. *PbBrA08^Banglim^*-1” was detected in the region of 18.86 to 38.86 cM with a peak position at 29.12 cM in the flanking marker “09CR.11390652”. The logarithm of odds (LOD) value was 74.8, and the phenotypic variance was 97.1%. *PbBrA08^Banglim^*-2” was located in the internal region of *PbBrA08^Banglim^*-1. The phenotypic variation was 26.0%, and the LOD value was 7.9 (Figure 3A, Table 2). The other analyses identified by inclusive composite interval mapping (ICIM) confirmed that these loci were significantly involved in the resistant phenotype, as indicated by high peaks in a similar position in which the commonly overlapped QTL region spanned ~1 Mb, corresponding to the physical distance, and encompassed 153 genes as determined using the reference genome. On the basis of a functional annotation, 11 genes (7 TIR-NBS-LRR class and 4 F-box) were considered as potential genes responsible for resistance in this genomic region (Table 3). 

### 2.4. Validation of QTL Based on the Expression, High-Resolution Melting (HRM), and InDel Marker Analysis 

To verify that *PbBrA08^Banglim^* is a novel locus, we compared the candidate gene sequence and expression levels with the previously reported three loci (*Crr1a, Rcr9*, *CRs*).

*PbBrA08^Banglim^* was detected on a region of A08 and encompasses several candidate genes considered to be potentially associated to the phenotype. Among these, BraA08g014480.3C (*Bra020861* in v1.5) was the candidate gene for *Crr1a* loci. The expression analysis by semi-RT-PCR showed that BraA08g014480.3C gene was not expressed in “09CR500” and “09CR501” genotypes both before and after infection, suggesting this gene did not have a role in resistance phenomena. Semi-RT-PCR of the *Crr1a* candidate gene confirms that it does not explain the resistance effect of *PbBrA08^Banglim^* in “09CR500” against the Banglim pathotype. 

We mentioned earlier in Section 2.3 that the *PbBrA08^Banglim^* locus contains the commonly overlapped QTL region (analyzed by WinQTL cartographer 2.5 and IciMapping 4.1), which spans ~1 Mb region. The investigation of sequence variation between the resistant inbred line “09CR-500” and susceptible reference genome “Chiifu” revealed that sequence variation occurred in 19,125 positions of which single nucleotide variation (SNV) = 15,739; deletion = 1,347; insertion = 1,873; replacement = 166. This region contains 11 candidate genes, of which two genes “BraA08g013190.3C” and ”BraA08g013630.3C” (Table 3) did not show any sequence variations between resistant and susceptible genotypes. Remaining 9 candidate genes showed 715 sequence variation sites. The primers for HRM analysis were designed to validate SNP variation in the *PbBrA08^Banglim^* locus, and genotyped with LightScanner System. For the InDel variation, the PCR amplicon was analyzed with LabChip^®^ GX Touch™. We designed a total of 34 primers spanning sequence variations from 9 genes. After genotyping among these, two markers—one SNP (“09CR.11390652”) and another InDel and (“09CR.11755754”) markers—were found to be closely linked to the *PbBrA08^Banglim^* locus (Figure 3). Further, genotyping with these two markers successfully distinguished resistant or susceptible phenotypes of DH population (Figure 5). These results suggested the potential use of these markers in marker-assisted selection program in Brassica crops for clubroot resistance.

## 3. Discussion

### 3.1. PbBrA08^Banglim^ Is a Stable Large-Effect QTL

In this study, we confirmed that the resistance of “09CR500” was controlled genetically by a single dominant gene based on the assessment of genetic inheritance in the DH population (Table 1). To dissect the clubroot resistance phenotype of “09CR500”, a genetic map was constructed. The total map length was 731.7 cM, containing 222 markers. Even though the number of markers was limited in the genetic map, it represented high genome coverage on the basis of genetic loci to physical positions, covering ~81.9% of the *B. rapa* reference genome. In particular, chromosome A08 had 95.2% coverage. Two robust QTLs, “*PbBrA08^Banglim^*-1” and “*PbBrA08^Banglim^*-2”, were detected on the A08 chromosome by two different methods (WinQTL cartographer 2.5 and IciMapping 4.1), which suggested that *PbBrA08^Banglim^* is a robust large-effect QTL involved in resistance (Table 2).

As a combination of resistant genome sequencing, we got the useful resources to develop markers, and the annotation information of genes in the QTL regions might be useful to interpret the resistant phenotype. Further, this data can be used as a resource to detect the resistance phenotypic plants.

### 3.2. RT-PCR and HRM Analysis Established the Novelty of the PbBrA08^Banglim^ Locus

*PbBrA08^Banglim^* locus was mapped on a region of A08; adjacent to this particular region, multiple loci have been reported. Initially, Hatakayema et al. characterized *Crr1a* loci from A08 chromosome, encoding the TIR-NB-LRR gene for clubroot resistance [23]. Subsequently, another QTL “*Rcr9”* was detected on A08 using genotyping by sequencing approach [27] and a new clubroot resistant locus *CRs* was recently identified and mapped on A08 chromosome by ddRAD-seq method [28]. These reports indicate that this region is likely a disease resistance cluster. Interestingly, although our *PbBrA08^Banglim^* locus mapped to A08 chromosome, it differs from already reported loci from the same chromosome which was confirmed by RT-PCR, HRM, and detailed comparative gene analysis. 

**Figure 4:**
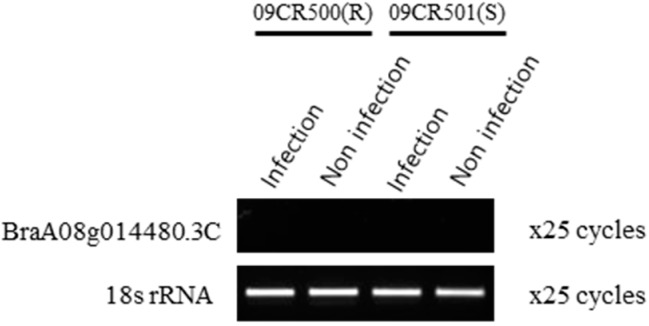
Expression analysis of the *Crr1a* candidate gene to validate that *PbBrA08^Banglim^* of resistant line “09CR500” is not same allele. Semiquantitative RT-PCR was done in resistant line “09CR500” and susceptible line “09CR501”. “Infection” refers to the treatment of pathotype “Banglim” to plants, and “Non infection“ indicates that no treatment has been performed. The *B. rapa* 18s rRNA was used as a control. PCR cycles are indicated on the right side. The candidate gene of *Crr1a* was not expressed in “09CR500” and “09CR501” in both conditions such as before and after infection.

*Crr1a* was first reported in A08, which indicated that *Bra020861* was a disease resistance gene by map-based cloning and characterized by transformation into Arabidopsis [23]. Therefore, to determine whether *PbBrA08^Banglim^* and *Crr1a* are similar or different, we performed a comparison of the “09CR500” genome sequence with G004 (*Crr1a*). A structural analysis demonstrated that “09CR500” encodes an NBS-LRR protein without the TIR domain, whereas *Crr1a* encodes TIR-NB-LRR protein [23]. Additionally, RT-PCR confirmed that the *Crr1a* candidate gene was not expressed in “09CR500” and “09CR501” before or after infection under the same conditions as the previous report [23] (Figure 4). A physical location comparison to the reference genome (V3.0) showed that marker “09CR11390652”, corresponding to *PbBrA08^Banglim^*, was located at 11.39 Mb, while a *Crr1a*-specific marker was located at 12.27 Mb (Table S6). These results confirmed that *PbBrA08^Banglim^* and *Crr1a* are not allelic. 

*Rcr9* was identified in “T19” using the genotyping-by-sequencing (GBS) method, and *Bra020936* was designated as a candidate gene by sequence variations between resistant and susceptible plants to the pathotype 5X [27]. Interestingly, *Bra020936* sequences (structure) in the resistant inbred “09CR500” genome were exactly the same as those in the reference genome Chiifu, which is susceptible to pathotype Banglim. This indicates that the *Bra020936* gene is not responsible for providing resistance in “09CR500” genotype. In addition, a separate experiment was performed in which an *Rcr9* locus-specific marker was used to assess “09CR500” and “09CR501”—a resistant and a susceptible type pool of DH population—and found that all of the samples were monomorphic and could not differentiate between resistant and susceptible phenotypes [28]. Overall, these observations indicated that *Rcr9* was not able to explain the resistance effect of *PbBrA08^Banglim^* in “09CR500” to the pathotype Banglim and confirms that *PbBrA08^Banglim^* is entirely different from *Rcr9* loci. 

**Figure 5:**
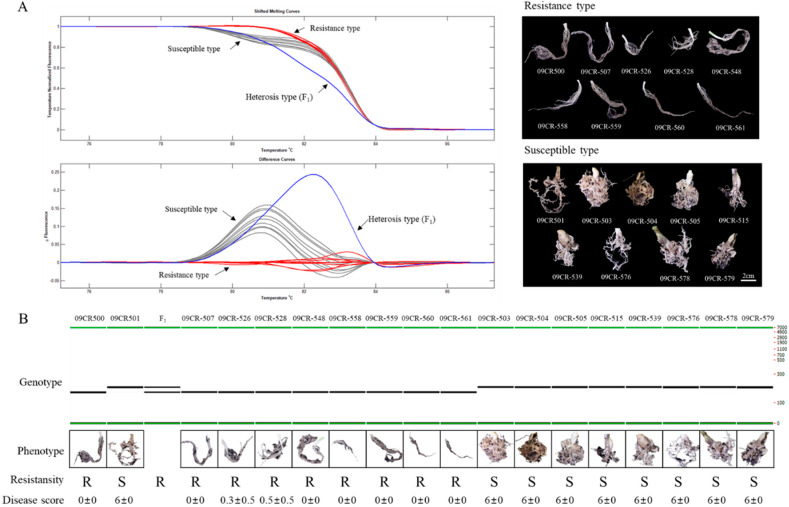
Single nucleotide polymorphisms (SNPs) and insertion and deletion (InDel) markers of the *PbBrA08^Banglim^* locus linked to clubroot resistance. (**A**) High-resolution melting (HRM) analysis of SNP marker “09CR.11390652” using the LightScanner System and phenotype. (**B**) Gel electrophoresis analysis of InDel marker “09CR.11755754” using LabChip^®^ GX Touch™ and phenotype. The plant genotype responses against Banglim pathotype are represented by R (resistant) and S (susceptible). Disease score was recorded as an average value with standard deviation.

*CRs*, recently identified using ddRAD-seq, demonstrated that *Bra020876* and *Bra020918* were candidate genes for resistance to the pathotype Seosan and their encoded proteins were functionally annotated as LRRs. Four HRM markers were developed and were able to distinguish resistant and susceptible genotypes among the population [28]. To confirm that *PbBrA08^Banglim^* loci is contrasting with *CRs* locus, we firstly used these four available markers. Among these markers, two SNPs were polymorphic in “09CR500” and “09CR501”, and further genotyping of the DH population revealed that *CRs* markers are located adjacent to the “09CR.11390652” marker (Figure 3). Secondly, the genotyping analysis using “09CR.11390652” markers was able to precisely distinguish all of the phenotypes of the DH population in response to infection with the Banglim pathotype, but the population could not be distinguished using *CRs* markers (Figure 3 and Figure 5); this indicates that *PbBrA08^Banglim^* is different from *CRs* loci along with confirmed utilization of “09CR.11390652” in marker-assisted selection in breeding program. The HRM-based evaluation effectively grouped resistant genotypes together and also distinguished susceptible and resistant genotypes. Our HRM genotyping data is consistent with phenotypic data, which supports the idea that resistance of “09CR500” was controlled by a single dominant *PbBrA08^Banglim^* loci (Figure 5, Table 1). Thirdly, to verify whether *PbBrA08^Banglim^* loci of “09CR500” genotypes behave the same or different to the Seosan pathotype (responsible for *CRs* loci) [28], a pathogenicity response of “09CR500” against two pathotypes Seosan and Banglim were evaluated and found to be contrasting in nature. The “09CR500” showed resistance to Banglim pathotype but was susceptible to Seosan [28]. Thus, the resistance of *PbBrA08^Banglim^* from “09CR500” was not allelic to *CRs*.

Overall, these results suggest that *PbBrA08^Banglim^* is a novel locus and “09CR.11390652” will be accurately used to distinguish resistant and susceptible cultivars of Brassica in plant breeding program. The development of resistant cultivars is one of the efficient ways to control clubroot disease, maintain sustainable agriculture, and to increase productivity of Brassica crops.

## 4. Materials and Methods

### 4.1. Plant Materials 

In this study, we used a double haploid (DH) mapping population (*N* = 81), named as “09CR-DH”, produced by microspore culturing and derived from a cross between “09CR500” and “09CR501”. Parent “09CR500” is a DH line derived from a progeny of a cross with ECD4 having a resistant phenotype to the local pathogen “Banglim”, which was classified as race 2 using Williams’ differential methods [46]. Line “09CR501” is susceptible to the pathotype “Banglim”, but it has the *CRb* loci, indicating a resistant phenotype to race 4 pathotypes as assessed by Williams’ differential methods [46].

### 4.2. Pathogen Inoculation and Evaluation of Disease Symptoms

The *P*. *brassicae* pathotype “Banglim” is a local pathotype that was collected by a seed company (BioBreeding Co., Anseong, Korea) from an area in Korea (local name, Banglim) having a high incidence of severe Clubroot disease. To retain the unique pathogenicity of the “Banglim” pathotype, it was propagated by continuously infecting common Chinese cabbage. The classification of this pathotype was presented as race 2, as assessed by Williams’ differential hosts sets [46].

Phenotypic evaluations were performed in greenhouses at Anseong and Deajeon in two years—2009 and 2012—using 81 DH lines, including the 2 parental lines. To investigate clubroot symptoms in plants, an inoculum was prepared as a spore suspension of 2 × 10^6^ spores/mL, as determined by counting with a hemocytometer. Then, 5-mL spore suspension was used to inoculate 2-week-old seedlings using the irrigation method (injection into the soil). The symptoms of individuals were assessed in roots at 6 weeks post inoculation. A new evaluation standard, dependent on the severity of gall (club) formation, was used. It consisted of seven scales from 0 to 6 as follows: 0 = no symptoms; 1 = very slight swelling, usually confined to lateral roots; 2 = a few tiny, separate globular nodules on the lateral roots; 3 = several small-sized nodules on lateral roots; 4 = large clubs on lateral roots joining together to form a lump, and slight swelling of main roots; 5 = larger clubs on main roots; 6 = severe clubs (lumps) formation all over roots, and complete change of root morphology (Figure 1). Host responses were assessed as “resistance” phenotype if the average scale value of the plant line was equal to or less than 2. 

To analyze QTLs, the scale values were converted to a disease index. It was calculated according to the following formula: disease index = [(*n*_1_ + 2*n*_2_ + … + 6*n*_6_)/*N*_T_ × 6] ×100, where *n*_2_ through *n*_6_ represent the numbers of plants with symptoms and *N*_T_ represents the total number of plants tested [9,47]. Additional phenotypic assays of the parental plants against pathotype “Seosan” were carried out at the Korean Research Institute of Chemical Technology following the protocols of Kim et al. [48] and Laila et al. [28]

### 4.3. Marker Development and Genotyping

We used previously reported single nucleotide polymorphic (SNP) markers that were used in map construction between “Chiifu” and “Kenshin” [49,50], and 106 newly developed primers targeting insertion and deletion (InDel) variations to construct a genetic map using the 09CR-DH population (Table S7). Additionally, some simple sequence repeat (SSRs) markers reported in Kim et al. [51] were used in this study to construct the genetic map frame. These marker sets were used as anchor markers to compare previous and current maps (Tables S7 and S2). To saturate the map, we performed “09CR500” genome sequencing with 30-fold Illumina sequence coverage using an Illumina Genome Analyzer-IIx system and paired-ends. To control sequence quality, low-quality reads were removed using fastqc. To ensure quality, the raw data were modified using the following steps: first, the adapter sequences in reads were deleted; second, reads that contained more than 50% low-quality bases (quality value ≤ 5) were removed. The clean reads were aligned to the reference genome using Bowtie2 [52], and then the BAM file was used to call SNPs and short InDel variations between the resequenced 09CR-500 and reference genomes using SAMtools and BCFtools [53,54]. The methods and parameters for identifying variations, such as SNPs and InDels, and designing the primers were previously reported by Pang et al. [49]. 

The nomenclature for the new markers followed the Bakus–Naur form described in Marcotty and Ledgard [55]: <3-letter institute code>_<single letter marker assay type designator>< MARKER ASSAY NAME>. The institute code “cnu” represents Chungnam National University, and the single letter marker assay type designators are those published by De Vicente et al. [56]. Those used here are “m” representing SSR, “s” representing SNP, and “i” representing InDel. 

The genotyping of SNPs was performed using two methods: the LightScanner System (Idaho Technologies, Salt Lake City, UT, USA), based on a high-resolution melting (HRM) analysis, performed in accordance with Pang et al. [49] and Li et al. [57]; and the Fluidigm^®^ EP1™ system using 96.96 Dynamic Array™ IFC (Fluidigm, South San Francisco, CA, USA), performed in accordance with Choi et al. [50], in which markers were named as <F-two digit code representing chromosome number>_<two digit code representing order of markers beginning to end on chromosome>. The PCR amplification was followed as an initial denaturation of 94 °C for 4 min, 35 cycles of 94 °C for 30 s, 55 °C for 30 s, and 72 °C for 30~60 s, followed by a final extension of 72 °C for 5 min. To perform comparisons with the previous loci, analyses of markers linked to previously identified Clubroot disease loci/genes (*Crr1a*, *Crr2*, *Crr4*, *CRc*, *CRk*, *CRs*, *CRa*, *Crr3*, *PbBa3.1*, *PbBa3.3*, and *CRb*) [13,22,23,28,58,59,60,61,62], which had already been mapped to the *B*. *rapa* A-genome chromosomes, were used to screen for polymorphisms in the parental lines (Table S8). The PCR amplification was followed as an initial denaturation of 94 °C for 4 min, 35 cycles of 94 °C for 30 s, the appropriate annealing temperatures for each primer pair for 30 s, 72 °C for 30~60 s, followed by a final extension of 72 °C for 5 min. PCR products were resolved by capillary electrophoresis using LabChip^®^ GX Touch™ (PerkinElmer, Waltham, MA, USA) and an HT DNA 1K LabChip^®^ kit (PerkinElmer, Waltham, MA, USA), or analyzed on 6% polyacrylamide gel electrophoresis. 

### 4.4. Genetic Map Construction and QTL Analysis

Linkage map construction was performed using JoinMap version 4.0 [63,64]. Linked loci were grouped using a logarithm of odds (LOD) grouping threshold, ranging from 2.0 to 3.0, and linkage groups (LGs) were assigned to 10 chromosomes. Locus order within the LOD groupings were generated for each LG using the following criteria: a recombination frequency smaller than 0.4 and an LOD larger than 1 for all marker pairs within each LG. Recombination frequencies were converted to centiMorgans (cM) using Kosambi’s method for map distance calculation [65], and the genetic map was drawn using MapChart [66]. 

For the QTL analysis, WinQTL cartographer 2.5 software using the composite interval mapping (CIM) was employed [67]. The walking speed for all QTLs was 1.0 cM, and the P-value inclusion threshold was 0.05. The threshold LOD scores were calculated using 1000 permutations [68,69]. To validate false-positive QTLs, we performed additional analysis with IciMapping 4.1 to detect putative QTLs [70].

To validate false-positive QTLs, we performed additional analysis with IciMapping 4.1 and confirmed that detection of QTLs is reproduced [66]. The missing phenotypic data were deleted using the “Deletion” command. For scanning of QTL, a step of 1 cM was used. The LOD threshold used to declare significant QTL was calculated using the 1000-permutation test with a type I error of 0.05 [68,69]. 

### 4.5. Annotation of Candidate Genes

To predict candidate genes in the QTL region, the putative functions of genes were obtained from the *Brassica* database (BRAD), which is based on genome version 3.0 [71]. The domains of candidate genes were annotated by performing a sequence analysis of the reference genome from the P-fam database using CLC genomic workbench 12.0.2 (CLC bio, Aarhus, Denmark) with a significance cutoff of 1.5. 

### 4.6. Conversion of Physical Location to BRAD Version 3.0

The SNP data information generated using BRAD v1.2 were further converted to the advanced reference genome v3.0 [71] to compare positions and annotations. The data were annotated to the reference genome v3.0 using binding sites of primer sets with CLC genomic workbench 12.0.2 (CLC bio, Aarhus, Denmark).

### 4.7. Semiquantitative RT-PCR Analysis

To confirm that the newly identified locus was novel and different from the adjacent resistance loci *Crr1*, *Rcr9*, and *CRs* [23,27,28], semiquantitative RT-PCR was performed. For reverse transcription (RT)-PCR, total RNA was extracted from plants using an RNeasy Plant Mini Kit (Qiagen, Hilden, Germany). Specifically, 2-week-old plants were inoculated with the Banglim pathogen and after 2 weeks, the roots were sampled. RNA quality was measured on a NanoDrop 2000 (Thermo Scientific, Wilmington, DE, USA). Then, 1 μg of total RNA was reverse transcribed using TOPscript™ RT DryMIX (dT18 plus) (Enzynomics, Daejeon, Korea) in accordance with the manufacturer’s instructions. The gene-specific primers were selected following the method of Hatakeyama et al. [23], and we used 18s rRNA gene-specific primers as a control [72]. The synthesized cDNAs were used as templates for PCR amplification with AccuPower PCR PreMix (Bioneer, Deajeon, Korea) and the following conditions: 95 °C for 5 min; 25 cycles of 95 °C for 20 s, 55 °C for 30 s, and 72 °C for 1 min; followed by a final extension of 72 °C for 5 min. Amplified PCR products were electrophoresed on a 1.5% agarose gel.

## 5. Conclusions

The prime aim of this study was to identify Banglim pathogen-specific resistant loci in *B. rapa*. To achieve this goal, we compared the candidate genes sequences of newly identified QTL regions with those of a previous report using whole-genome sequencing of resistant line “09CR500”. The QTL analysis mapped a novel locus *PbBrA08^Banglim^* for Banglim pathotype resistance on chromosome A08. This locus exhibited structural differences and expression variations compared with earlier reported QTLs, and the plant’s responses to the pathotype were also found to be different, suggesting that *PbBrA08^Banglim^* is a novel resistance loci to Banglim. The results of this study add to the available genetic information that can be used to elucidate the genetic mechanism of clubroot resistance. The newly developed marker “09CR.11390652” for *PbBrA08^Banglim^* will be beneficial in Brassica breeding programs for Banglim pathotype-specific resistance and gene pyramiding by allowing accurate phenotype selection.

## Figures and Tables

**Table 1:** Genetic analysis of clubroot resistance associated with “09CR500” in the doubled haploid (DH) population against the *P. brassicae* pathotype Banglim.

Location	Plant Materials	Total No.	Phenotype of			Expected Rtio (R:S)	χ^2^
			Resistance (R)	Susceptible (S)	Missing (-)		
Daejoen	09CR500	10	10	0	0	-	-
	09CR501	10	0	10	0	-	-
	DH population	81	40	40	1	1:1	0.01
Anseong	09CR500	10	10	0	0	-	-
	09CR501	10	0	10	0	-	-
	DH population	81	39	40	2	1:1	0.06

**Table 2:** Summary of QTLs detected for clubroot resistance against the Banglim pathotype of *P*. *brassicae* using composite interval mapping (CIM). LOD—logarithm of odds.

Loci Name	Location	Chr No.	Closest Marker	LOD	*R*^2^ (%)	Additive
*PbBrA08^Banglim^*-1	Daejeon	8	09CR.11390652	74.8	97.1	−47.0
*PbBrA08^Banglim^*-2	Anseong	8	09CR.11390652	7.9	26.0	−26.1

**Table 3:** Identification of related resistance genes within the *PbBrA08^Banglim^* region in *B*. *rapa*.

Gene ID		Gene Position (bp) ^a^		
v1.2	v3.0	Start	End	Protein Description ^b^
Bra020979	BraA08g013130.3C	11,388,577	11,391,460	Receptor-like protein/Leucine rich repeat
Bra020974	BraA08g013190.3C	11,427,768	11,428,159	LRR receptor-like serine/ threonine-protein kinase
Bra020945/ Bra020942	BraA08g013530.3C	11,658,233	11,659,423	F-box/kelch-repeat protein
Bra020936	BraA08g013630.3C	11,696,216	11,696,785	TIR domain containing disease resistance protein
Bra020928	BraA08g013730.3C	11,752,980	11,756,408	Leucine-rich repeat receptor-like serine/threonine-protein kinase
Bra020918	BraA08g013850.3C	11,809,250	11,815,278	LRR receptor-like serine/ threonine-protein kinase
Bra020901/ Bra020902	BraA08g014000.3C	11,921,301	11,924,248	F-box protein
Bra020876	BraA08g014350.3C	12,162,113	12,169,372	LRR receptor-like serine/ threonine-protein kinase
Bra020861	BraA08g014480.3C	12,271,553	12,276,276	Disease resistance protein
Bra020860	BraA08g014500.3C	12,289,217	12,290,401	F-box protein
Bra020847	BraA08g014610.3C	12,365,097	12,366,311	F-box protein

^a^ Physical location in *B*. *rapa* version 3.0. ^b^ Gene functions were assigned according to the best match of the alignments against Swiss-port databases (https://www.uniprot.org/) using BLASTP v2.9.0+ and Pfam database version 32.0 (https://pfam.xfam.org/).

## Supplementary Materials

The following are available online at https://www.mdpi.com/1422-0067/21/11/4157/s1, Table S1. Phenotypic trait values of parental lines and the DH population. Table S2. Marker information used to construct linkage map. Table S3. Summary of *B. rapa* genome sequencing data. Table S4. Distributions of total SNPs and InDels. Table S5. The *B. rapa* genetic map developed in the present study. Table S6. Clubroot linkage and marker information used on chromosome A08. Table S7. Primer information for this study. Table S8. Clubroot linkage and gene marker information used in the DH population.

## Abbreviations

QTL	Quantitative trait loci
DH	Doubled haploid
LG	Linkage group
SNP	Single nucleotide polymorphism
InDel	Insertion and deletion
LOD	Logarithm of odds
CIM	Composite interval mapping
ICIM	Inclusive composite interval mapping
TIR	Toll interleukin-1 receptor
NBS	Nucleotide binding site
LRR	Leucine-rich repeat
RT-PCR	Real-time polymerase chain reaction
