# Supplementary material for: Quantitative Trait Locus Mapping of Clubroot Resistance and Plasmodiophora brassicae Pathotype Banglim-Specific Marker Development in Brassica rapa
Source: Int J Mol Sci. 2020 Jun 10;21(11):4157. doi: 10.3390/ijms21114157 (PMC7312193; doi:10.3390/ijms21114157)
Supplement: Supplementary file 1 [file ijms-21-04157-s001.pdf]

Table S1. Phenotypic trait values of parental lines and the DH population (*N* = 81).

Location	Disease Index of Parents		Disease Index of DH Population	
	09CR500	09CR501	Average	Range
Daejeon	0	100	51.9 ± 47.6	0 - 100
Anseong	0	100	43.8 ± 42.2	0 - 100

Table S2. Marker information used to construct linkage map.

Maker ID	Type	Plants source [a]	Number of maker	Polymorphic rate (%)
cnu_s000-483 [b]	SNP	Chiifu, Kenshin	459	24.0
cnu_i001-032	InDel		32	68.8
F00-00 [c]	SNP		192	34.4
cnu_i222-383	InDel	Chiifu, 09CR500	106	62.3
BrID&EoE615 [d]	InDel	Chiifu	8	37.5
cnu_m000a, nia_m000a [e]	SSR		10	60.0

[a] The genome sequence of Chiifu (reference genome), Kenshin and '09CR500' were used to develop markers for the construction of linkage map. (reference genome v1.2, http://brassicadb.org/brad/index.php). [b] Pang et al. 2015; [c] Choi et al. 2020. [d] Wang et al.2011. [e] Kim et al. 2009

Table S3. Summary of *B. rapa* genome sequencing data.

Sample	Total No. of Raw reads (M)	Total No. of Raw Bases (G)	GC Rate (%)	Q20 Rate (%)	Q30 Rate (%)	Total No. of Clean Reads(M)	Total No. of Clean Bases(G)	Clean Date/ Raw Data(%)	Map Reads Rate(%)	Sequencing Depth
09CR500	202.58	25.32	39.74	95.88	92.19	201.11	25.14	99.29	94.10	87.06

Table S4. Distributions of total SNPs and InDels.

SNPs					InDels			
Homozygous	Heterozygous	Total	Syn CDS [a]	Non-syn CDS [b]	Insertion	Deletion	Total	CDS
1,867,179	534,016	2,401,195	11,510	28,200	301,252	294,426	595,678	44,291

[a] synonymous mutations in coding region; [b]non-synonymous mutations in coding region.

Table S5. The *B. rapa* genetic map developed in the present study.

Chromosome	Marker No.	Map length (cM)	Marker interval (cM)	No. of Markers				
				SNP	SSR	InDel	STS	SCAR
A01	6	29.3	4.9	4	2	0	0	0
A02	15	47.3	3.2	12	1	0	0	2
A03	24	82.5	3.4	17	5	1	0	1
A04	12	43.2	3.6	11	1	0	0	0
A05	15	108.0	7.2	14	0	0	1	0
A06	18	103.2	5.7	14	3	1	0	0
A07	13	68.6	5.3	12	1	0	0	0
A08	84	70.5	0.8	25	3	56	0	0
A09	21	131.2	6.2	19	1	1	0	0
A10	14	43.9	3.1	13	1	0	0	0
Total	222	731.7	4.4	141	18	59	1	3

Table S6. Clubroot linkage and marker information used on chromosome A08.

Maker ID	Marker Type	Locus Name	Primer Information (5' → 3')		Reference
			Forward Sequence	Reverse Sequence	
**09CR.11390652**	**SNP**	***qPbBrA08*** [banglim]	TCCGAGCCAAAAGAACAAG	CAACCCACAAAACACACCA	in this study
09CR.11755754	InDel		TCGCTCCAGGTACATAACT	ACTAACCATGTCAAACTGC	

BRMS088	SSR		TATCGGTACTGATTCGCTCTTCAAC	ATCGGTTGTTATTTGAGAGCAGATT	Suwabe et al. 2003 [1],
BRMS173	SSR	*Crr1*	CTTCTTCGATGGATTCAAGAGAAC	GAGGATGCAGTTGCTGTTGTT	Suwabe et al. 2006 [2]
crr1-Int2	InDel		TTTCCACATGATGATGAAGCTC	ATCGGGAATGTTCTCCAATCTA	Hatakeyama et al. 2013 [3]
CRs.A08.11344437			ATTCAGATGACCTTCTTCCGAC	GATATGTTACCACGGCGAAAAC	Laila et al. 2019 [4]
CRs.A08.12469094 *			GTTGGAGAACACACTGAGATCA	TCTCTCGAGGCTTCTGTTACTA	Laila et al. 2019 [4]
CRs.A08.11900241 *	SNP	*CRs*	ACAGCAAAACCTCTCTTCTCTC	ATGTCAAACAAGCGTCTAAAGC	Laila et al. 2019 [4]
CRs.A08.11941912 *			ACACTTAGGTGAAGAAGCAACA	GCATGCATCAAGTAACTTGCAA	Laila et al. 2019 [4]
CRs.A08.12169004			GAACTGGAGGCTTTTGAAACTG	CTTTGTCCTCAACTCCTGGAAC	Laila et al. 2019 [4]
Rcr9.Bra020936*	InDel	*Rcr9*	AGTGGAGAACCAAAGCCAAA	CAATTGTGGCCACCTTCTTC	Yu et al. 2017 [5] Laila et al. 2019 [4]

* Those markers were not polymorphic between '09CR500' and '09CR501'.

Table S7. Primer information for this study.

Converted Priemr ID	Original Marker ID	Chr	Primer Information(5'->3')		Start	End	SNP/InDel alleles
			Forward Sequence	Reverse Sequence			
cnu_s333	CKs_Br007977	A02	TGGTAAGCAGATTCCATCATCC	CGCTGGATTGTTTGCAGAAT	11,812,388	11,812,534	A/C
cnu_s334	CKs_Br008287	A02	CCGTGGGAAGATGTGTGGT	CATCATTCAATCGTTGCACACT	13,859,078	13,859,188	C/T
cnu_s335	CKs_Br008515	A02	ACATTCCGTTCCCGAGTCTT	TTTATCACAGCGGAGTTGGTG	15,831,367	15,831,516	G/A
cnu_s338	CKs_Br018449	A05	CAGCCTGTTGAATTGACGTG	AAGAAGTGCGGGGTTACGTT	21,458,674	21,458,809	G/A
cnu_s351	CKs_Br025524	A04	TGGTGAAGACAACAGCGACA	GGCCAGACGCAAGATCACT	10,652,214	10,652,360	A/C
cnu_s358	CKs_Br027245	A05	TGGAGTTGTGTTCAGCGTCA	TTCAGCAGCTCGTATGTCCA	23,143,750	23,143,885	C/A
cnu_s360	CKs_Br027667	A09	TGCTGTGTTTGTTCCGTAAGC	ATGACCACACCTGCGAGAAG	7,901,154	7,901,265	T/C
cnu_s361	CKs_Br027736	A09	CGTCGGTTTCTATCTCCGATG	AGCGAGAACTTCCCAAATAAGC	8,466,083	8,466,219	C/T
cnu_s371	CKs_Br029038	A03	GATACCCGTGTGCTCGTTTG	GGAATAGCAAAATAAGATGCTCAGG	6,209,966	6,210,069	G/A
cnu_s373	CKs_Br029179	A03	TCGGCTGTAGAATCGTCTATG	CAGACTGACGGTATGTGTTTGA	6,947,041	6,947,173	A/G
cnu_s374	CKs_Br029206	A02	AGAGAGATCGAATACAGACACCCTAA	GCTCAGATTCCGACCAGCTT	30,519,862	30,519,987	T/C
cnu_s390	CKs_Br030877	A08	TTGTTGGCTGAGAAATCGAAC	ATTTGGTGGAACCCAGCTTT	583,802	583,925	G/A
cnu_s392	CKs_Br031468	A01	CGGACATGAAACCAAATGGA	CCATATTGATCCGACCTGAAAA	18,820,717	18,820,860	C/T
cnu_s397	CKs_Br031705	A09	TACAAGCGCCCCTTTAGAGC	AAGCGCACACAAGATGGACT	43,132,495	43,132,626	T/C
cnu_s399	CKs_Br032337	A09	GAAGGAAGGAAGGAAGGATCAA	GTGCGATGTCTATGAGATGACG	27,868,096	27,868,220	C/T
cnu_s402	CKs_Br032938	A02	CCGTTTCAGACTATAATGTCCAAG	GACTTATCCCTACGTCCATGATT	25,938,322	25,938,441	G/A
cnu_s405	CKs_Br033127	A02	CGATAACATGCACACCCTCA	GCAATGTACCACCGACTGAA	17,499,923	17,500,072	A/C
cnu_s408	CKs_Br033797	A01	CAACCAACGCACTCTCTCAA	TCACCCTGTAAAACTCATCCTCA	14,775,033	14,775,177	C/T
cnu_s409	CKs_Br033814	A01	CCACGCCAGGGTCAGTAAAT	TTCTCACCGCAAATGCAAAT	14,935,560	14,935,665	T/G
cnu_s411	CKs_Br034542	A08	ATTCTCTCGGCTTCGCCTAC	TCAATTAACCACAGACCTGAAGC	13,597,676	13,597,823	G/A
cnu_s421	CKs_Br035848	A09	AAATGTGATGTGGCGTGAAA	TCAAAGTGCATCTCGTCAACA	4,485,155	4,485,259	C/T
cnu_s422	CKs_Br035952	A09	GGTTTCCTCTCTCTCCCTCTCA	TCTCCACAAGGCCATCTCTG	3,909,997	3,910,128	C/T
cnu_s423	CKs_Br036342	A02	AAGACTACACCCACCAGTTCCTC	ATCGACCAAGCATTCACCAC	25,767,354	25,767,467	G/A
cnu_s425	CKs_Br036406	A07	GGATAAGAACAGCCCGTCGT	CAGCGCAGAGAGAGTTGACC	4,424,236	4,424,369	A/G
cnu_s431	CKs_Br036771	A08	CAACCGGAGAATTGCTGAGA	GCTTCTTCTTTCCCCTTTGC	6,886,267	6,886,404	T/C
cnu_s432	CKs_Br036776	A08	GGAAAGAAAGAGACCTTTGTGGA	GACAACCGGGATGGAGTCTT	6,828,592	6,828,718	T/C
cnu_s438	CKs_Br037403	A09	GGTTGAGTGCCAAATCCTCA	TCTTCGCCTCTCACGTTGTT	552,856	553,000	T/C
cnu_s440	CKs_Br037563	A01	GAGAAAGAAAGGTTGGCCTGA	AGCTCACAGCAAGATCAGTGG	23,448,096	23,448,237	C/T

cnu_s441	CKs_Br037572	A01	GAAACTAAGGGTAGTGGCTGGA	AACTCTGACCAATACTCCCAACA	23,499,150	23,499,270	C/A
cnu_s452	CKs_Br038342	A02	TCATAACCCAAGTGAACGAAGA	GTTGGAGAAGATGATCCGTTTT	10,642,480	10,642,598	G/A
cnu_s456	CKs_Br038492	A01	GCAAATTCCATTTCGGCTTT	TCGGCTGTTATTCGAGAAGAGA	10,720,338	10,720,481	G/A
cnu_s457	CKs_Br038629	A06	TCCAAAATGGCAGCTTTCAG	ACCAGAAGAGGCAGGAGGAG	18,864,350	18,864,469	G/A
cnu_s460	CKs_Br038861	A01	ACTCGTGGCTGGAGGAAGAT	CGTCTCGCTATGGTTCGACT	12,822,741	12,822,875	G/A
cnu_s465	CKs_Br039199	A09	TCCCACCGTCCCTTTAGTTT	CGTCTTCCTCGTCCTCTTCA	39,255,068	39,255,182	C/A
cnu_s469	CKs_Br039475	A05	TTAGCGGGAGAAAGATGATATGAC	CTCGCTGGCTTCTTTGCTTA	27,286,508	27,286,629	T/C
cnu_s480	CKs_Br040509	A01	AATGCGTTTTGGCAGATGAG	TTTTGCGTGGCATGATGTT	29,569,525	29,569,648	C/T
cnu_s481	CKs_Br040520	A01	TCCCCTTAGGAAGGAAGTCAA	GTCCGTAACATCGCCAACAT	29,520,901	29,521,021	C/T
cnu_s482	CKs_Br040662	A05	ACAGTTCCATTCCGACAACG	TTCACTGCCTTGGATCTTCCT	12,624,939	12,625,063	C/T
cnu_i001	-	A08	GATGGATGATGTTCCTGAGTA	CTGTTTATCGATGACTTGGAC	15,107	15,431	D24
cnu_i002	-	A08	ATGGTGTGGTCAACTACAAAG	TGCTCACGGTACTTACAGTCT	225,608	225,976	I64
cnu_i003	-	A08	CATGTCTACGAAACATGCTCT	GTGATTGCAGGTAACATGACT	518,918	519,275	I22
cnu_i004	-	A08	GAGTTTCCGGTGAAAATAGAG	AGATCAAGGAATCTCAAGACC	765,243	765,577	I24
cnu_i005	-	A08	ACGAGTGTTACGAGAAGTTCA	TACTCACATAGCTTCGAGTGC	876,853	877,187	D52
cnu_i006	-	A08	TACCACGAAGACTACGACACT	GACCACACATCTATTCCTTTG	2,499,333	2,499,820	I183
cnu_i008	-	A08	GGATCTGACTGCTTGTGTCTA	ACAATCTTACTGCAACACACC	3,732,918	3,733,174	D28
cnu_i009	-	A08	ACAGAGAGACGATGAAACTCA	AGAGGAAACATGTGGAATAGAC	3,933,636	3,933,974	D26
cnu_i010	-	A10	TATGGTAGACCGAGCTGATAG	TCTTCAACAGCAGTAGGGTTA	6,763,973	6,764,296	D24
cnu_i011	-	A08	AGTTTCACAGACAAAGAGCTG	TCGTTTGGAGTTAGATACTGG	4,821,704	4,821,975	D48
cnu_i012	-	A08	CCTCTCTCTGACTATTGTTTCC	GAGCTATGGGAGAGTTCAGAT	15,799,912	15,800,244	D53
cnu_i013	-	A08	TATGGCCTTAATGGTACTGAG	AATCGTCCAAGGTAGTCTTCT	5,818,965	5,819,267	I21
cnu_i014	-	A08	GTTCCAATAGATCTTGCAGTG	CTGGTACTGGAACTGTCTCTG	5,553,530	5,553,863	I57
cnu_i016	-	A08	ACTCACCGGAGAGAGAAATAG	GAAGGAGAGGTTACGACAGAT	9,984,271	9,984,570	D32
cnu_i017	-	A08	GGCGGTTCTAATACATAACCT	TGCTTCTTCTCTTAGACCACA	8,456,134	8,456,356	D31
cnu_i018	-	A08	GCAGGTTTCTTCTCATCACTA	CCAATCATCTCTCAAGCTCTA	8,852,227	8,852,554	I26
cnu_i019	-	A08	CTGGAGAGATCCAATTTCAC	ACTGGATCTCGATATTTGCTC	12,272,957	12,273,325	I72
cnu_i020	-	A08	ATCTCTCATCTTCGTCTCCAT	AGAGTTGGAGAAGCTTAAGGA	16,089,895	16,090,150	D24
cnu_i021	-	A08	CAGGAAAAGAAGAAAGAGGAC	TTCTCACTGTTAGCGTTTACC	16,437,526	16,437,848	D57
cnu_i022	-	A08	AAGAACTCTAAGGAGGCTTGA	GAAACTTTGGTGTCTGATGTC	16,558,001	16,558,231	I36
cnu_i023	-	A10	TTGCTAGAAGACTCACCAAAG	TCACTGGGATTGAGAAGTCTA	17,152,047	17,152,260	D48
cnu_i024	-	A08	GTGGTCATGACTATGACTTGC	CTTGTAGTATGGAACCCAACA	17,863,951	17,864,248	D32
cnu_i025	-	A08	GCAAGTGAGCAAAGATAGAGA	TGTCCCTTGAGTAACTGAGAA	18,175,037	18,175,279	D54
cnu_i026	-	A08	GAACTCTTCTTCTCCAACGA	ATCACCGTCTTATCACCTTTC	18,722,548	18,722,818	I33
cnu_i027	-	A08	ACCATGAACGTAGTTCCAAA	TTATCTGATCAGTCGAAGAGG	18,959,194	18,959,499	I31
cnu_i028	-	A08	ATAGTTTGATCGCCTCTCTGT	GCAGCAGACTACAACTGAATC	19,334,834	19,335,110	D26
cnu_i029	-	A08	CTCTCTTTAACAACGGAGCTT	CAACTAACCTTTGGAAAACG	19,587,786	19,588,046	D62
cnu_i030	-	A08	AGAATATAGTCAACGGTGCTG	CTCTTCCTCATCATCCTCTCT	19,812,706	19,812,917	D24
cnu_i031	-	A08	ATCACCAGAACCACCACTAC	ACTCATGCGTACACCTATGTC	21,704,291	21,704,590	D36
cnu_i032	-	A08	GGACATTTCCCATAATCCTAC	CGGACCGGAGTAGATAGATAA	22,513,184	22,513,594	I84
cnu_i222	-	A08	TATCAGCAGCAGTCCAACCA	TACCAAAGGCACCGTCTCC	225,231	225,472	I21
cnu_i223	-	A08	ACGCCTTCTTGCCACCATA	GTCGCTGTTTCCGCTACACA	595,349	595,606	I15
cnu_i224	-	A08	CTTTCAATGGCGGCTAACTC	GCAGAACCTGGCGAATCTT	1,120,855	1,121,151	D18

cnu_i225	-	A08	TAGCCAAGCCAAAGTCAGCA	GCCTCGCATCCCACAGTTA	1,303,591	1,303,765	D42
cnu_i226	-	A08	ACTAACTCGGGCAGGCAAT	CAAGAGAGACAAATAAGTGGCGTA	2,239,425	2,239,695	I52
cnu_i227	-	A08	TCGTGTTACCTTGTCCTTCG	CCTATGCTTCCTCCTCTCTCA	6,764,018	6,764,173	D24
cnu_i229	-	A08	GGAGGTTGCTTCACAAACGA	CCCATCCGCTACAGGTCAT	5,698,921	5,699,154	D10
cnu_i230	-	A08	AATCCAGCCGTTTCTGTTCA	GCTTGGGAAGGAGTGATGC	9,098,346	9,098,539	D9
cnu_i231	-	A08	CGTCCTTCTCTTCCCATCTG	TACGCAGCAACACAACATCC	6,822,213	6,822,502	D24
cnu_i232	-	A08	ACTCATTTCGGATGGCACAG	CAGACCCAAGTTCCTGACAAA	7,871,080	7,871,278	D12
cnu_i233	-	A08	TCCGTTCACTATCAGGTGTGG	CAAGAGGGAGAAGCAGCAAA	8,852,346	8,852,584	I26
cnu_i234	-	A08	GAGAAGAAATCCCAAGGCTGA	AGTTCGCTGACGCTGTTGTT	18,298,358	18,298,523	D12
cnu_i235	-	A08	CGCCGAGATTTACTACAAGAATG	CATCAACACGAGGAGGACAAA	10,792,340	10,792,489	I22
cnu_i236	-	A08	GATACCGAAGGCGAAGGAGT	ATGACGACGGAGGAGAAACC	11,961,839	11,962,062	I15
cnu_i237	-	A08	GCTCTCTCTCTCTCTCTCTCTCTC	GGGCGTTTCGGTAAATGTT	13,127,034	13,127,199	I49
cnu_i238	-	A08	TGGTAAGGGTTGTCGTTCCT	GTGTTGGTGGTCATTGGTGA	13,766,064	13,766,328	I28
cnu_i239	-	A08	GGTCCAGTTCCATTCAGAGG	GGGATAGGTCGCTCCAGATA	15,791,258	15,791,540	D50
cnu_i240	-	A08	CTACTGTGGCAGCGAATCAA	AGGTGGCGTTATCTCAATCG	16,741,937	16,742,100	I27
cnu_i241	-	A08	TGACACTGCCTTGGACTGA	TGTTGTTGGAGGTTCTGAGG	17,152,088	17,152,232	D48
cnu_i242	-	A08	TGAGCAAGTGAGCAAAGATAGAG	AAGTTTCAAGAGCAACCGTCT	18,175,034	18,175,260	D54
cnu_i243	-	A08	CAACTAACTTCTGCCCACCAA	AATCCCGCCTCTGTAAATCC	19,587,891	19,588,018	D62
cnu_i244	-	A08	AGGCTTGTTCATTGGGATTG	AGCATTGTTTCCACCCTTGT	20,538,918	20,539,120	I81
cnu_i245	-	A08	TCCATCACCAGAACCACCAC	CATCTCCAACCCGATTCCAT	21,704,288	21,704,560	D36
cnu_i246	-	A08	GCGTTAGGAGGAGCGTTGA	TCCCGACAATGGCTTCAG	22,716,659	22,716,826	I6
cnu_i247	-	A08	ATGTGGAGAAACGAGCGGTA	TGCCGATAATGAAACAAGCA	22,873,105	22,873,329	D6
cnu_i305	-	A08	TGCCAACTCATCTCTGCTTACA	CCTCTGTCATCCACTCCATCA	11,162,923	11,163,074	D10
cnu_i306	-	A08	TCACACCTCCACTGAACTTGAT	GCCTTGTTTGGGCGTTTAT	11,171,167	11,171,388	D3
cnu_i307	-	A08	CTGCGAGAACGAGACCTACG	CTCCGACAACGACGAAGAAG	11,171,812	11,172,086	D9
cnu_i308	-	A08	TTTGATGAGGAATCGGGTTT	AAAGCAAGACGAGCAGGTTC	11,190,720	11,190,933	D20
cnu_i309	-	A08	ACCGTCTTCTCCTACTTTCAGC	AACTCTGCCACCATTCCATC	11,342,536	11,342,721	D15
cnu_i310	-	A08	ATTTGGCGATGGAAGTTTGT	AAGAGAGGAGAGAGAGCGATAGAT	11,475,023	11,475,177	D8
cnu_i311	-	A08	TCGGTTGCGGTATTTGTGTC	GCGATGGGTCGTATCTTTCTC	11,491,585	11,491,757	I23
cnu_i312	-	A08	CGTAGAAGAAACCAACGAAGAA	CGTCCGAGTAAACGAAGTGA	11,547,343	11,547,534	D33
cnu_i313	-	A08	ATGGCGGATGAGGGACAT	CATAGGAAGCACCGAGCAGT	11,636,111	11,636,374	D6
cnu_i314	-	A08	AACAACCACCACAACAGCAA	CCATTTCTCGCTTTCACCAC	11,673,672	11,673,907	D3
cnu_i315	-	A08	CACCAAGCAACACTGATTCG	GCGTGACCAGAAGGTTTAGG	2,484,472	2,484,737	I22
cnu_i316	-	A08	TCGGAGAAGAAGAAGGGAAGG	GCCTGAGAAATCGTCCATCG	11,853,505	11,853,675	D21
cnu_i317	-	A08	TGGCACCCACACACACATAC	GTCCTTTCAGCAAATCAACCTG	11,914,841	11,915,123	I35
cnu_i318	-	A08	GGACAGCGAAACACCCATC	TGGAACGAAGGCAGGTTG	12,247,350	12,247,516	D8
cnu_i319	-	A08	CCAATAGAGCCCATAAAGAACG	CAAAGCGAACGAGGGAGAC	12,324,835	12,325,100	I16
cnu_i320	-	A08	GGTTCTTTGCTCTGTTGTTCTCA	GTTAGGCATTGGCGTTTCC	12,418,543	12,418,823	D13
cnu_i321	-	A08	AGTGAACGACGAAAGATGATTG	AGAGACAAAGGGATAGGCTGTAA	12,543,525	12,543,777	D11
cnu_i322	-	A08	AGGAAGGAAGGTTTGAGAAGG	CAGCAACGGTAGTGGAACA	12,591,374	12,591,578	D40
cnu_i323	-	A08	TTGCCTGAGATGAAGAAATGG	GGATGATGCGTTAGGGTGAA	12,702,403	12,702,552	I23
cnu_i324	-	A08	AGAGGTGGGAGAGAGTCAAAGC	CCAATGAAACAACAAAGGCTGA	12,736,927	12,737,124	D32
cnu_i325	-	A08	CATAGCCACGACTTGATGCTT	GTTCCAATCCCACCACAGAC	12,751,069	12,751,325	I28

cnu_i326	-	A08	TTGAGGTGTGGTGTTTGTGC	TGGGTATCTCGTGTTGTGGA	12,954,699	12,954,968	D9
cnu_i327	-	A08	AAAGTCTCGTCTCCCATTGTTT	AGAATCATCAAGCATCCCAAC	13,122,336	13,122,542	D19
cnu_i328	-	A08	GCACAAAGGCCATAAACT	TCTTTAGGAGGGGGTATATT	281,098	281,255	I12
cnu_i329	-	A08	AAAGACAAAACTGGAGAAGG	CGGAAAGTGCTAGATTCAA	603,935	604,132	D13
cnu_i330	-	A08	TTCACCCACGCCTACTCA	ACAAACCCTAACCTTCAAG	1,110,105	1,110,235	I12
cnu_i331	-	A08	CCACGTCTCAAATCCTACA	CCATATTAGTCAAACGCATC	1,327,824	1,328,004	I15
cnu_i332	-	A08	GAGGCCACCATATATAAGA	AGGAGAGACAAGTAGAGAA	2,071,102	2,071,295	I13
cnu_i333	-	A08	TTTCTCTGTTCCTTACGGTT	TGGCTTGTCTGGTTGTTT	2,381,691	2,381,840	I14
cnu_i334	-	A08	CTACCATGATCACTTCCTCT	GCCCGCATTGATTTACTT	2,670,086	2,670,285	I14
cnu_i335	-	A08	ATGCGTTTAGTGTTCCAGT	CCCCTGCGAAAAATTTCC	2,947,435	2,947,600	D15
cnu_i336	-	A08	CCACCAATCCTATCGCTT	GATCTCAGCGCTTCTCCA	3,492,540	3,492,689	I10
cnu_i337	-	A08	GATTCTACCCTTCTGCAC	CATCGTCTTCTTCTTCTTCT	3,913,630	3,913,758	I15
cnu_i338	-	A08	TCCGCATTACTCTCTTCA	GCGAAATTAAGGAACCAC	4,344,988	4,345,118	I13
cnu_i339	-	A08	CCCGATTCTCCGCTATGA	GTGTCGATCCCTTGCTTT	6,270,123	6,270,318	I12
cnu_i340	-	A08	TACCTCAGCAGCTTGCAC	CTTATTGACGGAAGAACTTG	6,891,839	6,892,025	I15
cnu_i341	-	A08	TACCCATCTTTTCCAAACC	CTACTCTCTCTCTCCTCTC	7,241,431	7,241,610	I12
cnu_i342	-	A08	GGAAGGCGGAACACTGAA	ACACATAACGACCCGAGA	8,163,632	8,163,796	I10
cnu_i343	-	A08	TACACCAACCCTCTCCTC	CCTATACCACAACTTAATCC	8,792,021	8,792,122	D12
cnu_i344	-	A08	CCCCTTCAACTTTTCTCA	TCAGTTCCCCTTCTTCTC	9,071,979	9,072,074	I12
cnu_i345	-	A08	GTCAACATCACAAGGGAG	ACAAGGATCAAGTACAGCA	9,213,498	9,213,613	I12
cnu_i346	-	A08	CTGAGGCCAAAACTTGTT	ATCTGTTAGCTTTTCCCC	9,405,003	9,405,189	D13
cnu_i347	-	A08	GACTGCACGCATAATAAGA	GGTTTGAGGTGGTTTTATGA	9,992,757	9,992,881	I15
cnu_i348	-	A08	AGGGAGCTGAGAATGTGA	CCCTCTGTAAAACCAAAACC	10,117,737	10,117,926	I12
cnu_i349	-	A08	GTTTGTGTTGTGTGTGTGT	ATAAGTCGTTTTCCCTCGT	10,209,559	10,209,710	I12
cnu_i350	-	A08	CGTACCAAACAGACCATAA	TTCAAAAATTACGCGGGG	10,283,155	10,283,330	I13
cnu_i351	-	A08	GACAAGGGGCAACATAGAA	AAACGGTAAAGGCAGCAA	10,348,214	10,348,391	I12
cnu_i352	-	A08	TACGTCAAAGTCTACTGCT	CCTGAACAACAACCACAT	10,398,554	10,398,731	D12
cnu_i353	-	A08	CTAGCCCCAAACTCAACA	CTCCTTCGTCTTCCTTTC	10,607,325	10,607,523	D11
cnu_i354	-	A08	GAACCAGAGAGAGAGAGAG	ATCCAAAACAACCAAACCC	10,622,939	10,623,081	I13
cnu_i355	-	A08	GCTCTTTAACCTCTCGCT	CACACACGGCAAAATTATCA	10,679,792	10,679,912	D12
cnu_i356	-	A08	AATCCTCCTCGTCTATCA	CCTTCACCTTCACCTTCA	10,804,978	10,805,105	I12
cnu_i357	-	A08	AGTAAGCGTGAATGGAGA	AGCTGTGGCTATATGGGA	10,984,078	10,984,263	I18
cnu_i358	-	A08	TACACTTCACCTGACCAAC	ATCACTACTCCACTACTCC	11,921,714	11,921,849	I14
cnu_i359	-	A08	TTTCGGTTTGGGTTCACT	TCTTTAAATGCTCCACGCT	12,009,752	12,009,860	I15
cnu_i360	-	A08	GTGATAGTTGGTGTTCTGG	GAAGTGGTAGGAGTGAGG	13,055,593	13,055,789	I16
cnu_i361	-	A08	GGTCAGAAGGGAATATTCA	GGCATAGAGTAGAAGTACA	13,270,468	13,270,627	D15
cnu_i362	-	A08	AACAGGAAGAAAGGAGCGA	AAGAAACCGCCAACCAAA	14,267,854	14,268,003	I14
cnu_i363	-	A06	GTTTAGGTTTGTGAGTGTG	TAAAGTCAAAACCGCTCC	4,109,106	4,109,229	I14
cnu_i364	-	A06	CCTGAACCTCAAATCATCAA	ATCTACTCAACCACACAC	4,109,627	4,109,784	D12
cnu_i365	-	A06	TCTCCTCCTTACCTTTCTT	GAGACTTTCAGGGACATT	4,345,826	4,345,946	I12
cnu_i366	-	A06	GGCGCTCTATCAACAAAA	CACGATTGTTCTAGGTCT	4,454,457	4,454,587	I13
cnu_i367	-	A06	CATCCTGTTCGTTGCTTT	CGTCGCGTGATATAGATT	4,629,149	4,629,271	I12
cnu_i369	-	A06	TGAAGAAAGACGCACGAA	CCAAACTCGAATCCACAA	5,628,200	5,628,310	I15

cnu_i370	-	A06	TCAGTCAAAATCCCAGCA	TTCCCTTCTCTCTCTCTC	5,804,366	5,804,517	I13
cnu_i371	-	A06	GATTTTCAACGACCGGCT	AATAAACATGCCCCACCAC	5,869,100	5,869,209	I15
cnu_i372	-	A06	GCATAGCCAAATCCAACT	CCGGATTGAAGCGTTAGA	6,007,052	6,007,184	D12
cnu_i373	-	A06	TTATGTAGTTGCGGAGAG	GGAGTTAAAGGATCAAAGAG	6,071,594	6,071,752	I14
cnu_i374	-	A06	TGGCTTATCAGGTTTGGT	CTCTTGCGCTTTATCATC	6,072,563	6,072,656	I15
cnu_i375	-	A06	AGAGAAGAGAAGAGAGGAAG	GCGATCAAAAGGAATTGG	6,158,845	6,159,002	D13
cnu_i376	-	A06	GAGAAAAGGCAGAGGGTGA	CGTCAGGTAGATCTTGGT	6,463,278	6,463,437	I15
cnu_i377	-	A06	TTGATGTTTGTACTGGAGGA	ACAGAGAACCAGGAGAGA	6,522,104	6,522,246	D13
cnu_i378	-	A06	ACCAAATCATGTCGATTCC	CTTCCGCCACCTTTATTC	6,776,095	6,776,208	I14
cnu_i379	-	A06	CGGCGTTTAGGACATTTT	CATATAGTGGTGTCGCAT	6,824,169	6,824,288	I13
cnu_i380	-	A06	CCCTTCAACAATCATCAGACA	CCACCCATATCTTCCCTC	6,831,865	6,832,024	D12
cnu_i381	-	A06	GATAGCTAAAGTCCATTCC	CCACATCTCAAATATCACAC	7,025,355	7,025,488	D14
cnu_i383	-	A06	GCTAGTACATGTGTGATGA	GTGATGATGATGAGAGTG	8,729,011	8,729,139	D13

Table S8. Clubroot linkage and gene marker information used in the DH population.

Maker ID	Marker Type	Locus na me	Primer information (5' → 3')		Reference
			Forward Sequen ce	Reverse seque nce	
09CR.11390652	SNP	*qPbBr08*[banglim]	TCCGAGCCAAAAGAACAAG	CAACCCACAAAACACACCA	in this study
09CR.11755754	InDel		TCGCTCCAGGTACATAACT	ACTAACCATGTCAAACTGC	
BRMS088	SSR	*Crr1*	TATCGGTACTGATTCGCTCTTCAAC	ATCGGTTGTTATTTGAGAGCAGATT	Suwabe et al. 2003 [1],
BRMS173	SSR		CTTCTTCGATGGATTCAAGAGAAC	GAGGATGCAGTTGCTGTTGTT	Suwabe et al. 2006 [2]
crr1-Int2	InDel		TTTCCACATGATGATGAAGCTC	ATCGGGAATGTTCTCCAATCTA	Hatakeyama et al. 2013 [3]
CRs.A08.11344437	SNP	*CRs*	ATTCAGATGACCTTCTTCCGAC	GATATGTTACCACGGCGAAAAC	Laila et al. 2019 [4]
CRs.A08.12469094			GTTGGAGAACACACTGAGATCA	TCTCTCGAGGCTTCTGTTACTA	Laila et al. 2019 [4]
CRs.A08.11900241			ACAGCAAAACCTCTCTTCTCTC	ATGTCAAACAAGCGTCTAAAGC	Laila et al. 2019 [4]
CRs.A08.11941912			ACACTTAGGTGAAGAAGCAACA	GCATGCATCAAGTAACTTGCAA	Laila et al. 2019 [4]
CRs.A08.12169004			GAACTGGAGGCTTTTGAAACTG	CTTTGTCCTCAACTCCTGGAAC	Laila et al. 2019 [4]
Rcr9.Bra020936	InDel	*Rcr9*	AGTGGAGAACCAAAGCCAAA	CAATTGTGGCCACCTTCTTC	Yu et al. 2017 [5], Laila et al. 2019 [4]
TCR-079	SSR	*CRb*	TGACGTTCAATCAAAGCCTGA	TTTAGCAATCAAATGCAAATTCAA	Zhang et al. 2014 [6]
TCR-108	SSR		CGGATATTCGATCTGTGTTCA	AAAATGTATGTGTTTATGTGTTTCTGG	
B50-C9-FW/B50-RV	InDel	*CRc*	GATTCAATGCATTTCTCTCGAT	CGTATTATATCTCTTTCTCCATCCC	Sakamoto et al. 2008 [7]
HC688-4-FW/HC688-6-RV	InDel	*CRk*	TCTCTGTATTGCGTTGACTG	ATATGTTGAAGCCTATGTCT	
BRMS-096	SSR	*Crr2*	AGTCGAGATCTCGTTCGTGTCTCCC	TGAAGAAGGATTGAAGCTGTTGTTG	Suwabe et al. 2003 [1]
BRMS-100	SSR		CTCTTGAGAATCAGAGAGAGATTAC	GATCTTCATTATATTCATCTCTCTC	
BRMS-101	SSR	*Crr4*	TTGGTCGGTCACTCCTTATACTGAC	CAGGCGTTGCCTTTATGTACTCTAC	Suwabe et al. 2006 [2]
BRMS-125	SSR		GTTCTCAAAGGGAAACCGAAAAACA	GAGTTGGCCAGAGATTTACATGCGT	
sau-um231a	SSR	*PbBa3.1*	GATCCATCGTCGTCTCCAATAC	TAGTACTGCTTAAGCCCCTCTCC	Chen et al. 2013 [8]
sau_um438a	SSR		GTGTTTACGAAACAGAGTCGCC	GTGAGCATGTCTGAAGGAGAGAC	
cnu_m073a	SSR	*PbBa3.3*	TGGCATTGACAGAGCTAGTA	TTTATTTAGTTTCATACCCT	Chen et al. 2013 [8] Kim et al. 2009 [9]
cnu_m610a	SSR		ATGAAGGAGACATGGATGGG	GGTAATTCCGTAGGTTTTATTTAAGG	
sau_um398a	SSR		AATCTTCTAGGTCTCCTTCGCC	CTTTCTTCAGTCTCCTCTCTCCC	

References:

1. Suwabe, K.; Tsukazaki, H.; Iketani, H.; Hatakeyama, K.; Fujimura, M.; Nunome, T.; Fukuoka, H.; Matsumoto, S.; Hirai, M. Identification of two loci for resistance to clubroot (*Plasmodiophora brassicae* Woronin) in *Brassica rapa* L. *Theor. Appl. Genet.* **2003**, *107*, 997-1002.
2. Suwabe, K.; Tsukazaki, H.; Iketani, H.; Hatakeyama, K.; Kondo, M.; Fujimura, M.; Nunome, T.; Fukuoka, H.; Hirai, M.; Matsumoto, S. Simple sequence repeat-based comparative genomics between *Brassica rapa* and *Arabidopsis thaliana*: the genetic origin of clubroot resistance. *Genetics* **2006**, *173*, 309-319.
3. Hatakeyama, K.; Suwabe, K.; Tomita, R.N.; Kato, T.; Nunome, T.; Fukuoka, H.; Matsumoto, S. Identification and characterization of *Crr1a*, a gene for resistance to clubroot disease (*Plasmodiophora brassicae* Woronin) in *Brassica rapa* L. *PloS one* **2013**, *8*, e54745.
4. Laila, R.; Park, J.I.; Robin, A.H.K.; Natarajan, S.; Vijayakumar, H.; Shirasawa, K.; Isobe, S.; Kim, H.T.; Nou, I.S. Mapping of a novel clubroot resistance QTL using ddRAD-seq in Chinese cabbage (*Brassica rapa* L.). *Bmc Plant Biol.* **2019**, *19*.
5. Yu, F.; Zhang, X.; Peng, G.; Falk, K.C.; Strelkov, S.E.; Gossen, B.D. Genotyping-by-sequencing reveals three QTL for clubroot resistance to six pathotypes of *Plasmodiophora brassicae* in *Brassica rapa*. *Sci. Rep.* **2017**, *7*, 4516
6. Zhang, T.; Zhao, Z.; Zhang, C.; Pang, W.; Choi, S.R.; Lim, Y.P.; Piao, Z. Fine genetic and physical mapping of the *CRb* gene conferring resistance to clubroot disease in *Brassica rapa*. *Mol. breed.* **2014**, *34*, 1173-1183.
7. Sakamoto, K.; Saito, A.; Hayashida, N.; Taguchi, G.; Matsumoto, E. Mapping of isolate-specific QTLs for clubroot resistance in Chinese cabbage (*Brassica rapa* L. ssp. *pekinensis*). *Theor. Appl. Genet.* **2008**, *117*, 759-767.
8. Chen, J.; Jing, J.; Zhan, Z.; Zhang, T.; Zhang, C.; Piao, Z. Identification of novel QTLs for isolate-specific partial resistance to *Plasmodiophora brassicae* in *Brassica rapa*. *PLoS One* **2013**, *8*, e85307.
9. Kim, H.R.; Choi, S.R.; Bae, J.; Hong, C.P.; Lee, S.Y.; Hossain, M.J.; Van Nguyen, D.; Jin, M.; Park, B.S.; Bang, J.W. Sequenced BAC anchored reference genetic map that reconciles the ten individual chromosomes of *Brassica rapa*. *BMC genomics* **2009**, *10*, 432.
